# Close Follow-Up of Patients with Neurofibromatosis Type 1 Reduces the Incidence of Malignant Peripheral Nerve Sheath Tumour

**DOI:** 10.3390/cancers17081306

**Published:** 2025-04-12

**Authors:** Maria Pia Iasella, Dries Ruttens, Daphne Hompes, Vincent Vandecaveye, Raf Sciot, Christophe Deroose, Thomas Douchy, Thomas Decramer, Sandra Jacobs, Ellen Denayer, Frank Van Calenbergh, Eric Legius, Hilde Brems

**Affiliations:** 1 Centre for Human Genetics, University Hospitals Leuven, KU Leuven, 3000 Leuven, Belgium; 2 Department of Paediatric Oncology, University Hospitals Leuven, 3000 Leuven, Belgium; 3 Department of Paediatric Oncology, Princess Máxima Centre for Pediatric Oncology, 3584 CS Utrecht, The Netherlands; 4 Department of Surgical Oncology, University Hospitals Leuven, 3000 Leuven, Belgium; 5 Radiology Department, University Hospitals Leuven, 3000 Leuven, Belgium; 6 Department of Pathology, University Hospitals Leuven, KU Leuven, 3000 Leuven, Belgium; 7 Nuclear Medicine, University Hospitals Leuven, and Nuclear Medicine and Molecular Imaging, Department of Imaging and Pathology, KU Leuven, 3000 Leuven, Belgium; 8 Department of Neurosurgery, University Hospitals Leuven, 3000 Leuven, Belgium

**Keywords:** neurofibromatosis type 1, WB-DW/MRI, ANNUBP, MPNST

## Abstract

**Background/Objectives:** Neurofibromatosis type 1 (NF1) patients have an increased risk for benign and malignant neoplasms, leading to increased morbidity and mortality. Of these, malignant peripheral nerve sheath tumours (MPNSTs) are the most common malignant neoplasms causing death in NF1 patients. MPNSTs mostly originate from pre-existing plexiform neurofibromas (PNs). Many MPNSTs first pass through the premalignant stage of atypical neurofibromatous neoplasm with uncertain biologic potential (ANNUBP). With this study, we aimed to test whether active surveillance in adults with NF1 changes the natural history of MPNST development. More specifically, we wanted to evaluate the hypothesis that early detection of peripheral nerve sheath tumours (PNSTs) suspected of ANNUBP can lead to timely surgical removal of ANNUBPs, which in turn might reduce the incidence of MPNST in NF1 individuals. **Methods:** We retrospectively collected data on NF1 patients who were under surveillance with whole-body diffusion-weighted magnetic resonance imaging (WB-DW/MRI or DWI) and clinical exams at University Hospitals Leuven (UZL) or in one of the UZL network hospitals between 2012 and 2022 and who were 16 years or older at the time of their first WB-DW/MRI. The expected number of MPNST cases was calculated based on prior population-based studies, with statistical comparison using Poisson and Binomial distributions. **Results:** We included 276 patients with a total observation period of 1329.2 person-years. In total, 65 surgical interventions were performed in 58 individuals (21% of the cohort). WB-DW/MRI was followed by surgical intervention in 15.5% of the cases. We diagnosed 15 ANNUBPs in 14 patients. No cases of MPNST were observed against the expected 3.96 cases (*p* = 0.019). **Conclusions:** Active surveillance, incorporating WB-DW/MRI and timely surgical interventions for ANNUBP, significantly reduced the incidence of MPNST in this cohort.

## Simple Summary

Neurofibromatosis type 1 is one of the most common cancer predisposition syndromes, with affected individuals facing an increased risk of various benign and malignant tumours. Among these, malignant peripheral nerve sheath tumours are the leading cause of cancer-related mortality in NF1 patients. Complete surgical removal is necessary for a cure, but this is often unfeasible due to the tumour’s extensive growth and challenging anatomical location. Malignant peripheral nerve sheath tumours frequently arise from pre-existing plexiform neurofibromas, often progressing through a premalignant stage known as atypical neurofibromatous neoplasm of uncertain biologic potential. Unlike malignant peripheral nerve sheet tumours, these lesions can be effectively treated with surgical excision using minimal resection margins, preventing malignant transformation. Our retrospective study provides evidence supporting the benefits of strict surveillance through whole-body imaging. By enabling early detection and timely surgical intervention for premalignant lesions, this approach can significantly reduce progression to malignancy, ultimately improving both morbidity and mortality in these patients.

## 1. Introduction

Neurofibromatosis type 1 (NF1) is an autosomal dominant genetic condition with a birth incidence of one in 2000 to one in 3000 [1,2,3]. The disease is caused by germline loss-of-function variants in the *NF1* tumour suppressor gene at 17q11.2 [4]. *NF1* encodes the protein neurofibromin, a negative regulator of the RAS proto-oncogene, which is a key signalling molecule in the control of cell growth [5]. The disease is characterised by café-au-lait macules (CALMs), lentiginous macules in the axillary and inguinal region, iris Lisch nodules, multiple benign and malignant neoplasms and other features [6].

Malignancies associated with NF1 are high-grade intracranial glioma, malignant peripheral nerve sheath tumour (MPNST), breast cancer, gastrointestinal stromal tumour (GIST), and pheochromocytoma/paraganglioma (PPGL), with malignant glioma and MPNST having the highest malignant potential [7]. The incidence of cancer (including brain glioma) is higher in NF1 patients, and several cancers occur at a younger age compared to the general population. This is demonstrated in a Finnish cohort study showing a standardized incidence ratio (SIR) of 5.03 in NF1 patients, with the highest SIR values in children (including benign brain glioma: SIR 62.9 in children aged 0–14 years). In this cohort, the cumulative risk was 38.8% at 50 years of age, with a lifetime cancer risk of 59.6% (compared to 3.9% and 30.8%, respectively, in the general population) [8]. Furthermore, survival in NF1 patients with cancer is lower compared to matched controls [8]. This holds especially true for MPNST [7,8]. 

MPNSTs often originate from plexiform neurofibromas (PNs), which are a type of benign peripheral nerve sheath tumour (PNST) arising from single or multiple branches of nerves or nerve plexuses [9]. Some MPNSTs develop from plexiform neurofibromas through a premalignant stage of atypical neurofibromatous neoplasm with uncertain biologic potential (ANNUBP) [10]. Clinically, these tumours often show continuous growth over time and may cause pain. Further, they present an increased glucose uptake on fluorine-18-labeled fluorodeoxy-glucose (^18^FDG)-PET scan [11]. On MRI, an ANNUBP might present as a distinct nodular lesion (DNL) [12]. Histologically, these lesions are characterised by at least two of the following features: cytologic atypia, loss of neurofibroma architecture, hypercellularity, and a mitotic index >1/50 high-power fields (HPF) and <3/10 HPF [10]. Genetically, these tumours frequently show loss of the *CDKN2A/B* gene locus [13,14]. According to the 2024 proposed integrated consensus criteria for ANNUBP *CDKN2A/B* homozygous inactivation alone or heterozygous inactivation with at least one of the aforementioned histological features is sufficient for a diagnosis of ANNUBP [15]. MPNSTs histologically have features of ANNUBP but with a mitotic index of ≥10/10 HPF or 3–9/10 HPF combined with necrosis. Molecularly, the presence of *SUZ12/EED* inactivating mutation, *TP53* inactivating mutation, or significant aneuploidy (segmental gain or loss of at least eight different chromosome arms) is sufficient for diagnosis of MPNST [15]. The lifetime risk for developing an MPNST is 8–15.8% (compared to 0.003% in the general population), with a median age at diagnosis between 20 and 40 years [7,8,16,17]. The 5-year disease-specific survival remains poor, ranging from 31.6 to 60% [7,18]. Overall, MPNST is the most common malignant neoplasm causing death in NF1 patients [7,8].

PPGL and GIST are also more frequent in NF1 patients compared to the general population. Concerning PPGL, the lifetime risk is 1–5% with the highest prevalence during the fourth and fifth decade. Current guidelines do not recommend routine screening for PPGL [19]. GIST presents in NF1 patients most frequently during the fourth to seventh decade. It is estimated that a little over half of these tumours are asymptomatic, and the remaining half present with gastrointestinal bleeding, obstruction, abdominal pain, palpable mass, nausea, or weight loss [20,21,22]. A Japanese prospective cohort study showed a prevalence of 6.3% in NF1 individuals who are 30 years or older [23].

Taken together, NF1 patients are at risk of developing multiple neoplasms, leading to increased morbidity and mortality in these patients. In order to improve the clinical outcome of these patients, a surveillance program to detect NF1-associated neoplasms in adults is highly relevant. Early detection of tumours is recommended to minimize tumour-related morbidity and mortality. With this study, we aimed to test whether active surveillance in adults with NF1 influences the incidence of MPNST. Early detection of PNSTs suspected of ANNUBP can lead to further investigation, resulting in timely surgical removal of ANNUBPs, which, in turn, can reduce the incidence of MPNST in NF1 individuals.

## 2. Materials and Methods

### 2.1. Patients

Basic surveillance of adult NF1 patients at our centre consists of a clinical visit with history taking and physical exam every two to three years, whole-body diffusion-weighted magnetic resonance imaging (WB-DW/MRI) at the age of 16–18 years (or later if not yet performed) and an annual breast MRI for women between 30 and 50 years. If WB-DW/MRI shows a tumour suspected of ANNUBP, an FDG-PET scan is performed. A tumour suspected of being ANNUBP based on clinical, radiological, and PET findings is removed surgically, if safely possible, using nerve-sparing surgery.

We retrospectively collected data on patients with a clinical or genetic diagnosis of NF1 who were under surveillance with WB-DW/MRI and clinical exams at University Hospitals Leuven (UZL) or in one of the UZL network hospitals between 2012 and 2022. The clinical diagnosis of NF1 was established in accordance with the revised diagnostic criteria [6]. All patients 16 years or older at the time of their first WB-DW/MRI were included. The follow-up time was calculated starting from the date of the first WB-DW/MRI that took place after 1 January 2012. We considered the last follow-up before 31 December 2022 or death as follow-up endpoints. The data collection regarding the information on demographics, medical events (occurrence of PN, ANNUBP, MPNST, PPGL, and GIST), and interventions was performed by a single person to ensure reliability under the supervision of two MDs. The date of a medical event or intervention was noted when available. If only the month was known, we used the first day of that month. If only the year was known, we used the first day of that year. Data collection was conducted in compliance with the declaration of Helsinki. Approval for this study was given by the ethics committee of UZ Leuven (MP021514 and S69071). Data were encrypted, and access to the data was restricted to individuals directly involved in the study. Informed consent was not required since the study concerns retrospective data collection. All data were treated confidentially by the investigators.

### 2.2. Whole-Body MRI

Three Tesla WB-DW/MRI (Ingenia, Philips Healthcare, Best, The Netherlands) was performed with parallel radiofrequency transmission and phased-surface coils. Free-breathing WB-DWI was acquired in the transverse plane at b0 and b1000 s/mm^2^ in 4 stacks from head to pelvis. The scanner software created apparent diffusion coefficient (ADC) maps from the DWI data. All DWI image sets were reformatted to coronal and transverse WB images. Correlative anatomic imaging consisted of free breathing coronal WB short tau inversion recovery fat-suppressed T2-weighted turbo spin-echo sequence acquired in 3 stacks and free breathing/dual breath-hold single-shot turbo spin-echo T2-weighted sequences acquired in 4 transverse stacks (Table S1). Anatomical images were also reformatted to WB images. No intravenous contrast material was administered.

WB-DW/MRI images were assessed by radiologists experienced in abdominal and oncologic imaging (V.V.). Characterization of PNSTs was based on T2-weighted (tumour size, margin, perilesional oedema, presence of split fat, fascicular and target sign) and DWI characteristics (b1000 signal intensity and ADC with mean threshold of 1.15 × 10^−3^ mm^2^) [24]. Adrenal gland tumours and GIST were similarly identified and characterized using T2-weighted and DWI characteristics.

### 2.3. [^18^F]FDG-PET/CT Scanning and Quantitative Analysis

[^18^F]FDG-PET/CT scans were performed with Siemens Biograph 16 HiRez, Siemens Truepoint 40 (Siemens Healthcare, Erlangen, Germany), or GE Healthcare Discovery MI4 (GE Healthcare, Chicago, IL, USA). Patients fasted for a minimum of 6 h before intravenous administration of 3–4.25 MBq [^18^F]FDG/kg body weight. Three hours after [^18^F]FDG administration, a high dose CT scan (85 mAs for Siemens and 50–450 for GE (modulated); 120 kV) with intravenous and oral iodinated contrast was performed, immediately followed by a PET scan (vertex to mid-thigh, or until feet if clinically indicated). The CT data were used for attenuation correction of the PET images. Scan acquisition was performed according to The European Association of Nuclear Medicine (EANM) procedure guidelines for tumour imaging and reconstruction parameters compliant with EANM Research (EARL 1.0) recommendations [25].

Image analysis of [^18^F]FDG-PET/CT was performed using hybrid orthogonal viewer (Hermes Hybrid Viewer (Hermes Medical Solutions, Stockholm, Sweden) or MIM Software (MIM Software Inc., Cleveland, OH). Maximum Standardized uptake values (SUV_max_) were calculated within each lesion. SUV_max_ values > 5.0 were considered of concern for (pre-)malignant transformation, and SUV_max_ values > 10 were considered highly suspicious for MPNST [26,27,28].

### 2.4. Pathological Definitions and Genetic Analysis

The diagnosis of PN, MPNST, PPGL, GIST, and glioma was based on the criteria defined by WHO panels [29,30,31]. Diagnosis of ANNUBP was based on the latest consensus criteria [15]. If clinically necessary, PNs, ANNUBPs, and MPNSTs were further analysed by array CGH, next-generation sequencing on an Illumina platform using Sanger sequencing and MLPA (MRC Holland).

### 2.5. Statistical Methods

The expected number of MPNST cases per year was calculated using the data from Uusitalo et al. (2016) [8]. *p*-values for both Poisson and Binomial distributions were calculated.

## 3. Results

### 3.1. Demographics

We included 276 individuals, of which 158 were women (57.2%) and 118 were men (42.8%), with at least one WB-DW/MRI and subsequent clinical and/or radiological follow-up. The total observation period was 1329.2 person-years with, respectively, 760.6 and 568.6 person-years in women and men. Age at last observation ranged from 16 to 77 years (mean 37 years, median 34 years).

### 3.2. Whole-Body MRI

In total, 419 WB-DW/MRI scans were performed, with an average of 1.5 scans per individual. Of the total cohort, 88 patients (31.9%) had more than one WB-DW/MRI.

### 3.3. Interventions

In total, 65 surgical interventions were performed in 58 individuals, 21% of the cohort. WB-DW/MRI was followed by surgical intervention in 15.5% of the cases. The surgical intervention and subsequent histopathological examination led to the identification of 15 ANNUBPs in 14 people (age range 16.2–60.7 years; mean age 30.4 years, median age 26.4), 14 secretory pheochromocytomas (age range 23.0–56.5 years; mean age 39.0 years, median 38.2), 5 GISTs (age range 35.2–68.8 years; mean age 53.5 years, median 53.9 years), 1 brain tumour (age 18.4 years) and 28 benign neurofibromas of different types. One desmoid tumour of the small intestine and one lymph node consistent with Castleman disease were removed surgically.

### 3.4. Tumours Detected During the Study Period

Table 1 shows the numbers of the tumours detected by WB-DW/MRI (ANNUBP, MPNST, PPGL, GIST, and brain tumour), specifying whether the tumour was diagnosed as a result of the surveillance program or not. Recurrences of tumours initially detected and treated before the surveillance period were excluded from this table.

During surveillance, a total of 321 PNs were visualized on WB-DW/MRI. This corresponds to a total of 152 people (55%) of the cohort, with an average of 2.11 lesions per person (average in complete cohort: 1.16 lesions; range 0–9). Of these lesions, 220 were previously known, and 101 were newly discovered. Table 2 shows the number of PNs, either previously known or detected through active surveillance, according to the anatomic site.

In total, 15 ANNUBPs were removed (age range 16.2–60.7 years, mean age 30.4 years, median age 26.4 years) as a consequence of active surveillance. In all but one, there was a suspicion of ANNUBP based on pain, clinical or radiographic appearance/growth, and/or PET scan. Eight lesions met the criteria for ANNUBP as per the 2017 consensus histologic criteria. Through molecular analysis, we could detect *CDKN2A/B* deletion in another seven lesions. As per the 2024 proposed integrated consensus criteria, the total number of ANNUBP cases is 15 [15]. Molecular analysis was not systematically performed for all lesions as this was performed on clinical indication. An FDG-PET scan was performed prior to surgery in all but three patients, and every pictured ANNUBP showed increased FDG uptake. We observed no MPNST and expected 3.96 cases of MPNST when accounting for age using the Finnish population data (*p* = 0.019, Poisson, *p* = 0.019, Binomial distribution) [8]. Table 3 shows the person-years follow-up with the expected and observed number of MPNSTs per age group.

In total, 5 GISTs (age range 35.2–68.8 years; mean age 53.5 years, median 53.9 years) were discovered through active surveillance. Two more GISTs were discovered outside routine clinical visits because of new symptoms (abdominal pain/diarrhoea and melena, respectively; last MRI 44 months and 9 months before diagnosis, respectively).

Fourteen secretory pheochromocytomas (age range 23.0–56.6 years; mean age 39.1 years) were diagnosed through active surveillance. There were no interval pheochromocytomas.

### 3.5. Follow-Up

At the last follow-up, 72 individuals were considered tumour-free, 199 had one or more tumours, and 5 were deceased (all of them males). Of the 199 persons with a tumour, 1 had a potential pheochromocytoma, and 4 had a lesion suspected to be ANNUBP. These people did not undergo surgery either because the operation was planned after the moment of data collection because of the high risk of postoperative neurological deficit or because caregivers and patients opted for a “watchful waiting” approach. The deceased individuals died of the following causes: cholangiocarcinoma (age 45 years), progressive brain stem tumour (age 39 years), oral carcinoma (smoking-related, age 60 years), sudden death (age 46 years) and unknown cause but unrelated to oncological problems (age 35 years).

## 4. Discussion

In this retrospective study, we show that one-fifth of the patients underwent surgery as a direct result of active surveillance. WB-DW/MRI was followed by an intervention in 15.5% of the cases, leading to the diagnosis of a (pre)malignant lesion in over half of these cases.

In the retrospective cohort, we were able to demonstrate a significant decrease in the incidence of MPNST when compared to the expected incidence based on population data from Finland, supporting the hypothesis that the detection and timely removal of PNSTs suspected of being ANNUBP can prevent further transformation to MPNST. We calculated the expected number of MPNST cases in our cohort using data from Uusitalo et al., 2016 [8], which, to our knowledge, represents the most reliable population-based data currently available. In contrast, our cohort comprises a university hospital-based population, potentially introducing a bias towards more severely affected patients.

In our cohort, a PN was present in 55% of the patients, corresponding to the upper end of the reported prevalences in the literature [32]. A total of 15 ANNUBPs were removed. Eight lesions met the criteria for ANNUBP as per the 2017 consensus histologic criteria. As per the proposed 2024 integrated consensus criteria, we could classify an additional seven lesions as ANNUBP because of *CDKN2A/B* deletion in combination with one histological criterion. We do not have an explanation for the high proportion of females with ANNUBP in our cohort (12/15; 80%). In a previous study of 63 patients with atypical neurofibromas, 31 females were reported (49%) [33]. The five cases of GIST were also females. A cohort study of 95 adult NF1 individuals in Japan showed GIST in 2 females and 4 males [23].

Currently, a combination of clinical information (growth in adulthood, pain), MRI-scan findings (growth in adulthood, nodular lesion), and PET-scan findings (FDG-avidity) are used to aid in the differentiation between ANNUBP and PN [10,34]. Further research is needed in order to expand the knowledge of existing biomarkers to predict the potential of ANNUBPs to transform into MPNST. An important first step in this direction would be to improve the definition of MRI imaging modalities and to identify the difference in FDG-PET characteristics between PN, ANNUBP, and MPNST in order to more effectively distinguish these conditions through imaging. Tissue biopsy can give valuable information but presents some important limitations, such as the often difficult accessibility of the lesions and heterogeneity within the tumour. The latter issue can be partly remedied by PET-guided biopsy [35]. The addition of *CDKN2A/B* deletion to the diagnostic criteria will improve the accuracy of the classification of ANNUBP. In our study, the incorporation of these molecular features led to the classification of seven additional tumours as ANNUBPs, which would not have met the criteria otherwise [10]. Likewise, pathological discrimination of ANNUBP from MPNST can be difficult. Also, here, molecular features of early MPNST can help discriminate these lesions from ANNUBP [15]. Regarding biomarkers, liquid biopsy would be an interesting approach, but this is currently poorly explored in the setting of ANNUBP and looking for copy number alterations might have little additive value to discriminate ANNUBP from early MPNST. The possibility of fragmentomics on cell-free DNA seems more promising [36]. A recent study demonstrated the biological role of ENG (endoglin) in MPNSTs and its potential role as a liquid biopsy biomarker and therapeutic target [37]. A similar approach for ANNUBP would be of great interest.

It is known that PNs have heterogenous growth behaviour, with frequently the central part of the lesion showing malignant degeneration. It has also been shown that some lesions might show spontaneous regression [38,39]. Also, some MPNSTs originate from PNs without going through an observable ANNUBP stage [33]. Currently, there are no good predictors for this tumoral behaviour [40]. Determining the appropriate frequency of clinical visits and imaging, as well as identifying the age range with the highest risk for malignant transformation, remains a key research priority. Most centres will remove ANNUBPs when feasible, provided there is no significant risk of serious surgical complications. However, the ability to accurately distinguish between ANNUBPs with a high versus low risk of malignant transformation would help avoid unnecessary interventions. This is particularly important given the potential burden of over-treatment, which can lead to financial strain on both patients and healthcare systems, as well as unnecessary treatment-related adverse effects from interventions that may not have been essential. Developing predictive markers for malignant transformation would enable a more personalized surveillance and treatment approach, ensuring that only those at the highest risk undergo surgical intervention while minimizing unnecessary procedures and associated burdens.

Since 2020, Selumetinib, a MEK1/2-inhibitor, has been FDA-approved for the treatment of symptomatic, inoperable PN in children with NF1 [41]. The drug negatively affects cell proliferation in NF1-deficient cells, reducing tumour burden and proving particularly effective in decreasing the size of PNs [42]. A recent study shows promising results on symptomatic PNs in NF1 adults and a broader-scale clinical trial has recently been published showing promising results also in adults [43]. Researchers have also begun exploring the potential of MEK inhibitors in the treatment of MPNST, though results have been mixed [44,45]. Future research should explore whether patients undergoing or having completed Selumetinib treatment develop ANNUBP and MPNST at the same rate as those who have not received the treatment. Additionally, it would be valuable to assess whether administering this drug (alone or in combination with drugs targeted towards other pathways) in cases of ANNUBP could prevent or delay the development of MPNST, potentially widening the therapeutic window for intervention.

Through this screening strategy, we diagnosed and treated 14 cases of secretory pheochromocytoma detected by WB-DW/MRI, followed by urinary catecholamine and metanephrine excretion analysis in cases with abnormal adrenal glands. There were no interval tumours reported. These findings demonstrate that WB-DW/MRI is also effective for the detection of PPGL, possibly decreasing the rates of morbidity and potential mortality associated with the condition. Two GIST cases were not discovered through the screening program. Although the number of patients is low, this finding suggests that follow-up through WB-DW/MRI does not allow for the detection all cases of GIST.

A limitation of this study is its retrospective nature, and prospective studies are needed to confirm the potential benefits of close surveillance on tumour management in adolescents and adults with NF1. Prospective studies are also needed to improve the diagnosis of the different types of PNSTs and their malignant potential using imaging modalities, including MRI with ADC, DWI, chemical exchange saturation transfer imaging [46], and FDG-PET. We also need to further study the added value of cell-free DNA fragmentomics, single-cell RNA sequencing, and spatial transcriptomics of tumour biopsies. The ultimate goal would be to identify those individuals that would benefit from nerve-sparing surgery or targeted drug therapy to prevent imminent malignant transformation using an individualised surveillance programme for adolescents and adults with NF1.

## 5. Conclusions

This study demonstrates that a surveillance program consisting of regular clinical examination and WB-DW/MRI followed by further investigations, if necessary, results in the detection of premalignant PNSTs, preventing further malignant transformation of these tumours. Most importantly, we could demonstrate a significant reduction in the number of MPNSTs in our retrospective cohort. This supports the approach for surgical removal of ANNUBPs to prevent evolution towards MPNST. Further prospective studies to optimize existing biomarkers and to develop new biomarkers are needed to help clinicians select the patients for nerve-sparing surgery at the optimal time point. The role of MEK-inhibition and other targeted treatments on natural tumour progression needs to be explored, as surgery for PN/ANNUBP can be associated with high morbidity.

## Figures and Tables

**Table 1:** Tumours observed.

Tumour Type	Male	Female	Total
GIST			
Interval tumours ^a^	0	2	2
Cases detected through surveillance ^b^	0	5	5
Total	0	7	7
PPGL			
Interval tumours ^a^	0	0	0
Cases detected through surveillance ^b^	7	7	14
Total	7	7	14
ANNUBP			
Interval tumours ^a^	0	0	0
Cases detected through surveillance ^b^	3	12	15
Total	3	12	15
MPNST			
Interval tumours ^a^	0	0	0
Cases detected through surveillance ^b^	0	0	0
Total	0	0	0
BRAIN TUMOUR			
Interval tumours ^a^	4	1	5
Cases detected through surveillance ^b^	0	1	1
Total	4	2	6

^a^, ^b^: Tumours were considered detected as a consequence of the surveillance program if diagnosis was made through physical exam/imaging performed at one of the regular clinical visits. If the tumour was diagnosed during the surveillance period but not on a scheduled visit, it was considered an interval tumour.

**Table 2:** Number of PNs.

Location	Cases in Previous Medical History	Cases Detected Through WB-DW/MRI	Total
Head and neck	50	16	66
Arms	39	10	49
Thorax	39	12	51
Abdomen	27	15	42
Pelvis	13	16	29
Legs	52	32	84
Total	220	101	321

**Table 3:** Cumulative incidence of MPNSTs in different age groups.

Age Group (Years)	Person-Years Follow-up	C.I. °	Expected MPNST per Year/Group	MPNST = 0 *p*-Value Poisson/Binomial
10 < x ≤ 30	627.6	0.085	0.0043/2.67	
31 < x ≤ 50	487.4	0.123	0.0021/1.01	
51 < x ≤ 80	214.2	0.158	0.0013/0.29	
Total	1329.2	-	0.0030/3.96	0.019/0.019

° C.I.: cumulative incidence till 30, 50, and 80 years.

## Data Availability

The data that support the findings of this study are included in the article. Further inquiries can be directed at the corresponding author.

## Supplementary Materials

The following supporting information can be downloaded at: https://www.mdpi.com/article/10.3390/cancers17081306/s1. Table S1: Acquisition protocol.
