# Supplementary material for: Close Follow-Up of Patients with Neurofibromatosis Type 1 Reduces the Incidence of Malignant Peripheral Nerve Sheath Tumour
Source: Cancers (Basel). 2025 Apr 12;17(8):1306. doi: 10.3390/cancers17081306 (PMC12025940; doi:10.3390/cancers17081306)
Supplement: Supplementary file 1 [file cancers-17-01306-s001.zip › cancers-3541971-supplementary.pdf]

Table S1. Acquisition Protocol.

	DWI			T2-weighted TSE	
	**Transverse**	**Coronal**	**Sagittal**	**Coronal**	Transverse
**Image stations head to mid-thigh**	4	MPR	MPR	5	4
**Sequence**	Single-shot SE-EPI			Multi-shot TSE	Single-shot TSE
**Respiration**	Free breathing			Free breathing / respiratory triggering*	Free breathing / dual breath-hold*
**Fat suppression**	STIR (TI = 250 ms)			STIR (TI = 200 ms)	none
**b-values (s/mm^2)**	0-1000			-	-
**Parallel imaging factor**	2,5			3	2,4
**Repetition time (ms)**	8440			7895 (1858*)	1002 – 1123
**Echo time (ms)**	67			80	180
**Slice thickness (mm)**	5	3	3	6	6
**Intersection gap (mm)**	0,1			0,6	0,6
**Slice number**	50/station			34/station	41/station
**Field of view (mm)**	420 x 332			250 x 462	375 x 340
**Acquired voxel size (mm)**	4.57 x 4.68			1.49 x 1.68	1 x 1
**Reconstructed voxel size (mm)**	2.19 x 2.19			0.96 x 0.96	0.73 x 0.73
**Number of signal averages**	1			1	1
**Acquisition time (min:sec)**	3:23 per station			2:15 (1:19*) per station	0:46 (0:35*) per station

DWI, diffusion-weighted imaging; SE-EPI, spin-echo echoplanar imaging; TSE, turbo spin-echo imaging; STIR, short T1 inversion recovery; SPAIR, spectrally adiabatic inversion recovery; * used only for thorax and abdomen stations.
